# The Thackeray Prize

**Published:** 1840-10

**Authors:** 


### THE THACKERAY PRIZE.

The prize of fifty pounds placed at the disposition of the Provincial Medical Association, by Dr. Thackeray, of Chester, was awarded, at the late meeting at Southampton, to Dr. William Davidson of Glasgow, for the best essay "On the Causes and Mode of Propagation of Continued Fever."

We are happy to have it in our power to announce to our readers, that the author has kindly placed this very valuable essay at our disposal, and it will appear as a Supplement to our next Number.

